# Local heat generation during screw insertion into diaphyseal bone: a biomechanical study on different conditions (e.g. screw type, material, mode of insertion)

**DOI:** 10.1186/s12891-021-04723-6

**Published:** 2021-09-30

**Authors:** Berit Paul, Andre Frank, Michael J. Raschke, Dirk Wähnert

**Affiliations:** 1 Department of Trauma, Hand and Reconstructive Surgery, University Hospital Muenster, Albert-Schweitzer-Campus 1, Building W1, 48149, Muenster, Germany; 2 Department of Trauma Surgery and Orthopedics, Protestant Hospital of Bethel Foundation, University Hospital OWL of Bielefeld University, Campus Bielefeld-Bethel, Burgsteig 13, 33617, Bielefeld, Germany

**Keywords:** Screw insertion, Thermal necrosis, Heat generation, Screw material, Screw design, Insertion technique

## Abstract

**Background:**

The implantation of screws is a standard procedure in musculoskeletal surgery. Heat can induce thermal osteonecrosis, damage the bone and lead to secondary problems like implant loosening and secondary fractures. The aim of this study was to investigate whether screw insertion generates temperatures that can cause osteonecrosis.

**Methods:**

We measured the temperature of twenty human femur diaphysis in a total of 120 measurements, while screws of different material (stainless steel and titanium alloy) and different design (locking and cortex screw) were inserted in three different screwing modes (manual vs. machine screwing at full and reduced rotational speed) with 6 thermocouples (3 cis and 3 trans cortex). Each was placed at a depth of 2 mm with a distance of 1.5 mm from the outer surface of the screw.

**Results:**

The screw design (cortical > locking), the site of measurement (trans-cortex > cis-cortex) and the type of screw insertion (hand insertion > machine insertion) have an influence on the increase in bone temperature. The screw material (steel > titanium), the site of measurement (trans-cortex > cis-cortex) and the type of screw insertion (machine insertion > hand insertion) have an influence on the time needed to cool below critical temperature values. The combination of the two parameters (maximum temperature and cooling time), which is particularly critical for osteonecrosis, is found only at the trans-cortex.

**Conclusion:**

Inserting a screw hast the potential to increase the temperature of the surrounding bone tissue above critical values and therefore can induce osteonecrosis. The trans-cortex is the critical area for the development of temperatures above the osteonecrosis threshold, making effective cooling by irrigation difficult. It would be conceivable to cool the borehole with cold saline solution before inserting the screw or to cool the screw in cold saline solution. If possible, insertion by hand should be considered.

## Background

The success of an osteosynthesis in trauma and orthopedic surgery depends on numerous factors. Some of them can be influenced by the surgeon and the technique used. Drilling holes and placing screws is an essential step in most osteosynthesis. This happens so often and routinely that most surgeons are not aware of effects on bone. In particular, the possibility of thermally induced bony necrosis during screw insertion might be underestimated.

The heat generation during several surgical procedures (e.g. drilling) has the potential to irreversibly change and therefore weaken the mechanical competency of the bone and could be one factor leading to damage such as screw and implant loosening resulting in construct failure, re-fracture, and delayed union or malunion [1, 2].

Bone tissue necrosis occurs immediately, if the temperature of 60 °C is exceeded [3]. At lower temperatures, the exposure time has a crucial influence on the occurrence of irreversible damage to the bone tissue. Whereas ten minutes at 45 °C cause thermal necrosis, only one minute at 47 °C is necessary to induce an irreversible damage. A heat of 50 °C causes necrosis after 30 s [3–5].

Drilling is a process that has already been extensively investigated with regard to heat development and optimization. It was shown, that under special conditions a temperature of 70 °C was exceeded [6]. The following factors have been identified to have an impact on the heat development during drilling: design and size of the drill bit, force applied to the drill, the speed of the drill, the application of a coolant, the sharpness and the times of reuse of the drill bit [7–10].

While Augustin et al. were able to demonstrate that the optimal method of drilling to generate as little heat as possible was the use of lower drill speeds and higher feed rates [11], there have been few studies to date that deal with factors influencing the temperature development during screwing in human bone.

Therefore, the aim of this study is to investigate if osteonecrotic temperatures can be induced by the screw insertion. Additionally, different conditions and their influence on temperature generation have been examined. Hence, the screw material, the screw design and the screwing mode have been investigated in a standardized method.

## Methods

### Study design

We measured peak temperature and duration while screws of different material (stainless steel and titanium alloy) and design (locking and cortical screw) were inserted into the human femoral diaphysis in three different screwing modes (manual and machine screwing at full and reduced rotational speed), for a total of 120 measurements. Each single combination of parameters was tested 5 times.

### Specimens

Ten pairs of fresh-frozen human femoral diaphysis (length 15 to 20 cm) were used. The mean age of the donors was 77.4 years (range 60–93 years). Six of the donors were female, four male.

### BMD analysis

Bone mineral density (BMD) of the cortical bone was determined for all specimens within the diaphyseal region by means of a peripheral quantitative computer tomography (pQCT). Therefore, all specimens were packed under vacuum and thawed to room temperature. CT scan with a slice thickness of 0.63 mm was performed (SOMATOM Emotion 6 CT scanner, Siemens Healthcare GmbH, Erlangen, Germany). Image data given in Hounsfield unit (HU) was calibrated to volumetric BMD (vBMD) values given in mg hydroxyapatite per ccm (mgHA/ccm) using a BMD phantom (Model. No. 8783219, Siemens AG, München, Germany). The BMD was measured within a region of interest (ROI) over 12 slices.

### Implants

**Fig. 1:**
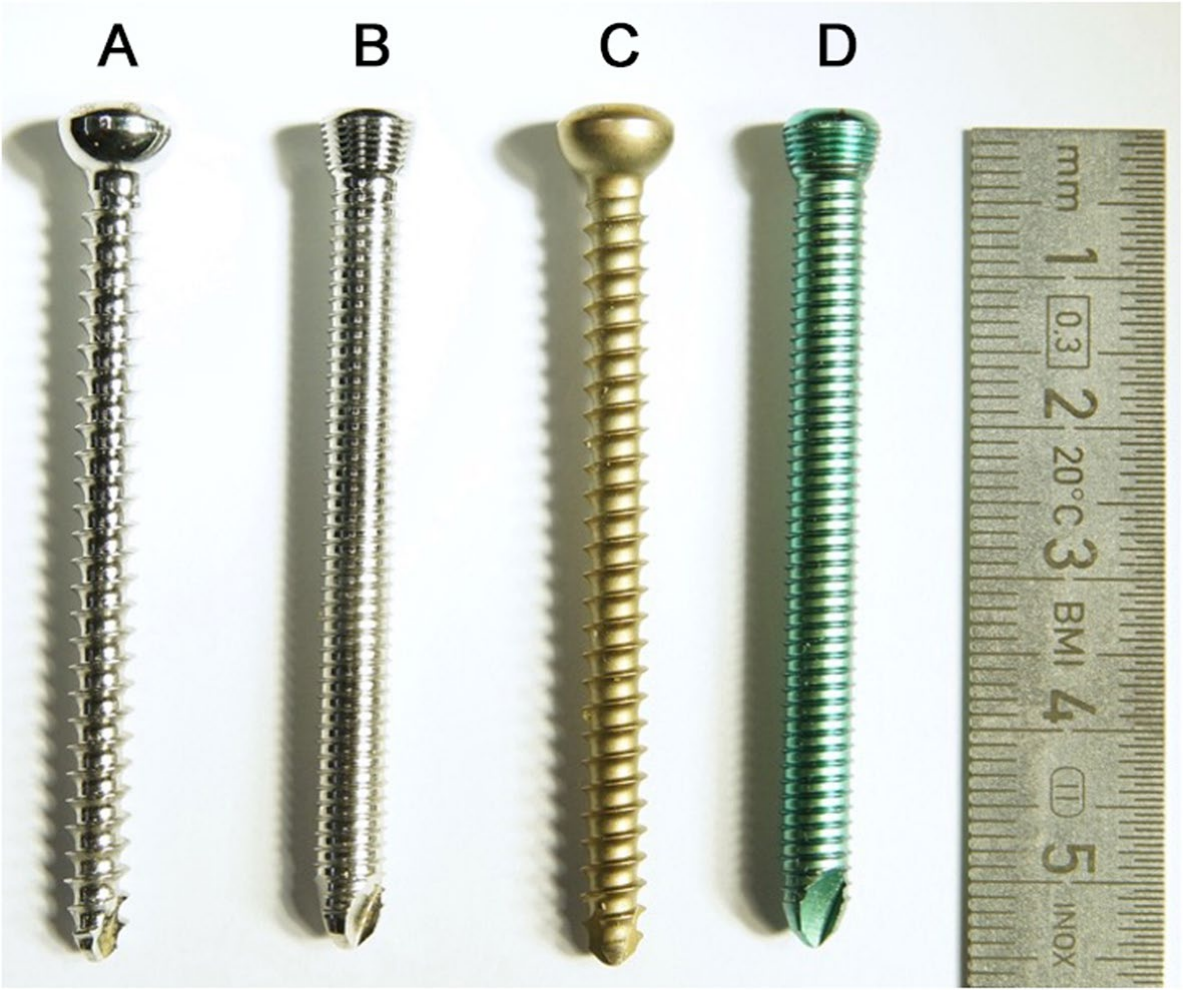
Photograph taken by the authors showing the screws used for this investigation. All screws had a length of 56 mm. **A** cortex screw steel, 4,5 mm **B** locking screw steel,5 mm **C** cortex screw titanium, 4,5 mm **D** locking screw titanium, 5 mm

30 × 5 mm head locking screws (15 made of titanium alloy and 15 made of steel) and 30 × 4.5 mm cortical screws (15 made of titanium alloy and 15 made of steel) were used for this investigation (Fig. 1; DePuy Synthes, Zuchwil, Switzerland). All screws had a length of 56 mm. Each screw was used for two measurements, which allowed us to perform 120 tests in total with 60 screws. In a preliminary test, no statistically significant difference between the first and second screwing process of the same screw could be found.

### Specimen preparation

Specimens were thawed at room temperature and then fixed in a standard bench vise. First, six screw holes per femur diaphysis were pre-drilled following standard surgical recommendations using 4.3 mm (locking screws) or 3.2 mm (cortical screw) drill bits. For all drilling operations we used a commercially available non-surgical cordless screwdriver (Fa. Würth - Model BS 18A Compact, Künzelsau, Germany).

In order to consider the working length of each screw, we measured the cortical thickness for the cis- and the trans-cortex at each drill hole using a depth gauge prior to inserting the screw. Additionally, all parameters (material, design, screwing modes) were examined on one pair of specimens in order to minimize the influence of bone density/thickness as far as possible.

**Fig. 2:**
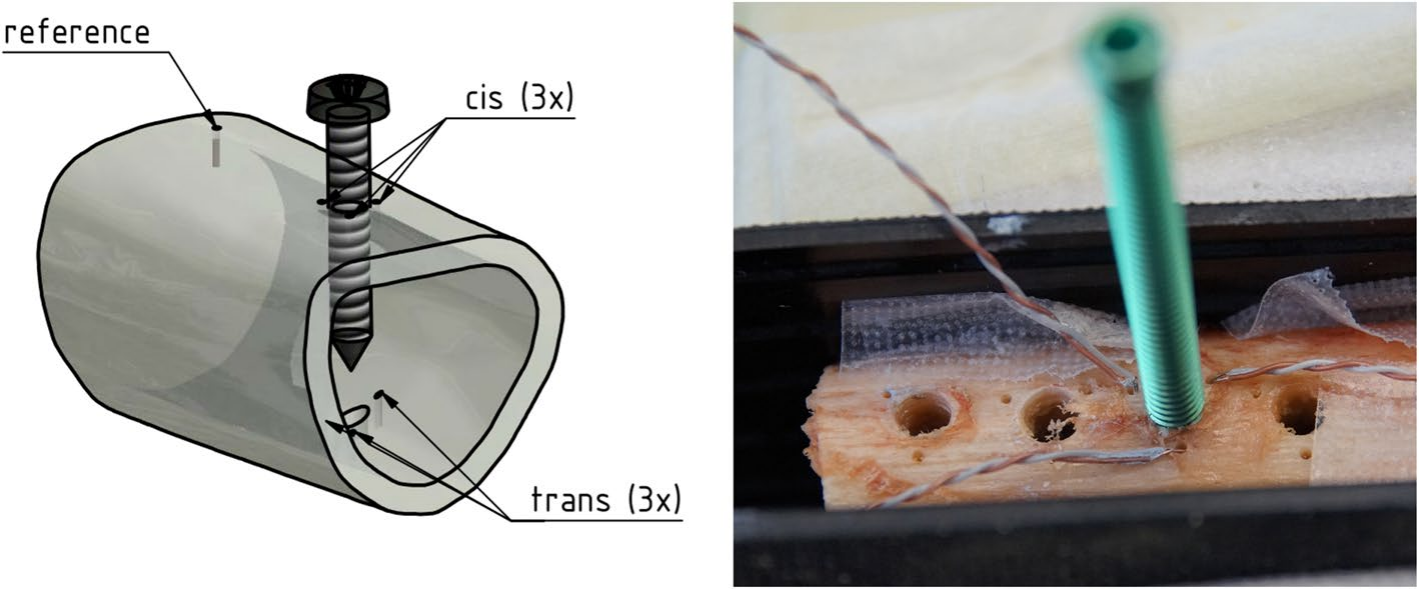
Photograph taken by the authors showing a specimen with pre-drilled screw holes and prepared holes for the thermocouples. Within one screw-hole a titanium locking screw is placed surrounded by three thermocouples in the cis- and three in the trans-cortex

To measure temperature near the screw hole, a custom-made jig was inserted into each of the pre- drilled screw holes. It was thus possible to drill six 1 mm holes around each screw hole (three at the cis- and three at the trans-cortex). These holes had a depth of 2 mm and were placed at a distance of 1.5 mm from the screw outer surface (Fig. 2).

After preparing the bones as described above, we implanted six PFA-isolated thermocouples (Fa. TC Direct, Mönchengladbach, Germany Typ T- Nr. 401–304, ∅0,02 mm) into the 1 mm pre-drilled holes and adapted them with “Arctic MX-2 Compound” insulation gel to ensure temperature conductivity. A seventh sensor was placed next to the bone as a reference value with the room temperature.

### Temperature measurement

All measurements were performed at room temperature. Three screwing modes were tested on each bone: manual insertion, full speed and reduced speed machine drilling. For manual insertion a standard surgical screwdriver was used. For insertion with the drilling machine, standard compressed air-operated surgical drilling machine was adapted, so that a standardized free-running speed could be guaranteed with the help of a rotary wheel. Two different power settings (688 rounds per minute (rpm) and 787 rpm; the compressed air line was adjusted to 8 bar) were used to simulate a full speed insertion as well as a reduced speed insertion.

Before each measurement, the correct position of the thermal sensors was checked. Each individual test lasted for 120 s. By placing the sensors, it was possible to derive the temperature at the cis- and the trans-cortex separately.

To calculate the cooling time until the temperature increase falls below 10 Kelvin, the measured temperature was mathematically interpolated (linear range in the cooling phase), and the resulting time determined. For this purpose, only the values of the sensors with a temperature rise of more than 10 K were used and averaged for further calculation. Under physiological conditions (body temperature 37 °C), the evaluated cooling time means the time required for the bone tissue to fall below the temperature of 47 °C [2–5]. To estimate which parameters have particularly high risks of exceeding osteonecrotic thresholds, we combined the parameters (maximum temperature and cooling time) and tightened the limits even more (maximum temperature increase 15 K) and a necessary cooling time of more than 70 s. Thus, we can safely assume osteonecrotic values [2–5].

### Modified temperature coefficient for bones

**Fig. 3:**
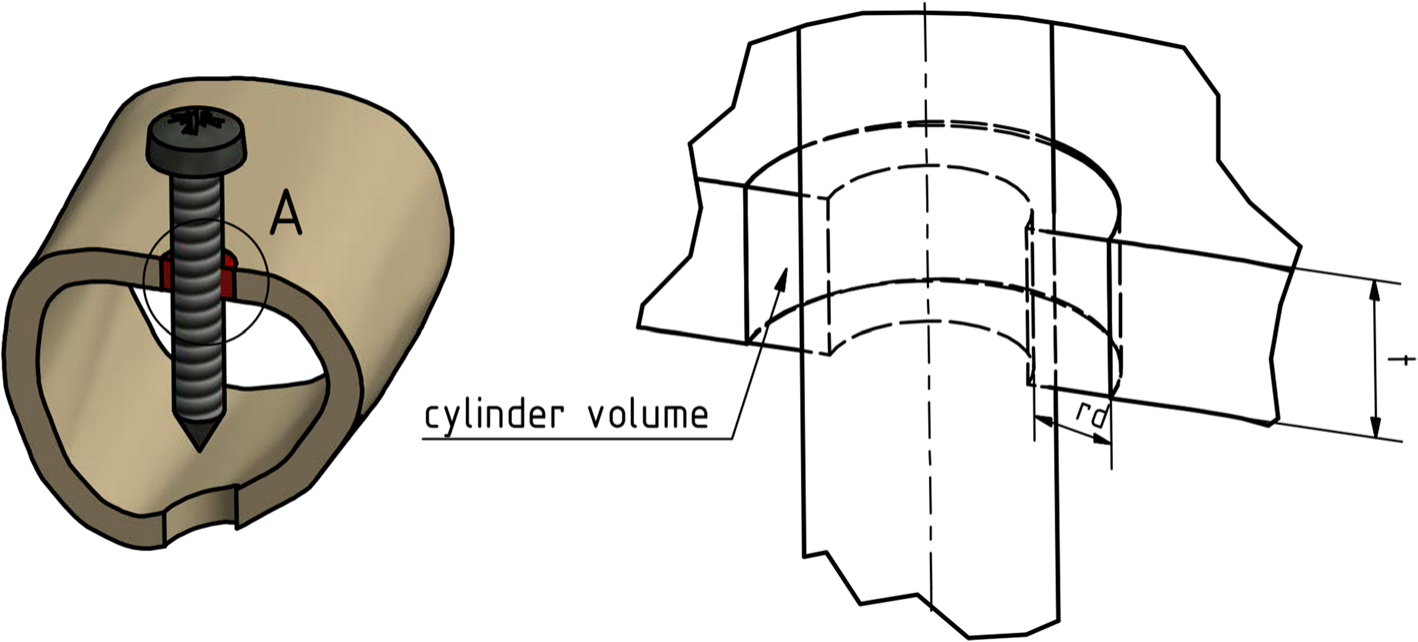
Schematic illustration created by the authors (using Inventor Professional 2016, Autodesk, San Rafael, United States) for the calculation of mTCb

In addition, a modified temperature coefficient for bones (mTCb) was calculated, which allowed us to investigate the temperature development in relation to the density of the tested bone. The coefficient is based on the assumption that there is a correlation between the temperature development and the amount of bone(mass) around the predrilled canal (Fig. 3). The coefficient describes the temperature increase in Kelvin relative to 1 g bone mass.

$$mTCb=\frac{\Delta T}{mass}=\frac{\Delta T}{BMD\times cylinder\ volume};\left[\frac{K}{g.}\right]$$

Using the BMD and volume of the cylinder (radial distance (rd) and cortical thickness (t)), the mass of the region of interest can be calculated.

The influence of the variation in bone density and cortical thickness is thus eliminated and statements can be made independently, e.g., on the material of the screw.

The mTCb does not describe the temperature gradient over a wider area in the bone.

### Data acquisition and evaluation

With the implemented 7 thermocouples the temperature while screw insertion was recorded using a 8 channel thermocouple data logger by Pico Technology (Pico Technology, Cambridgeshire, United Kingdom). The software used for data recording was the PicoLog-Recorder (Pico Technology, Cambridgeshire, United Kingdom, Windows, Release 5.21.5).

The temperature was recorded in degrees Celsius with a frequency of 10 Hz. For each side (cis- and trans-cortex), an average of the three sensors/cortex was used in order to approximate the average temperature increase. In the event that the screw directly touched a thermocouple, this sensor was excluded.

Additionally, for all measurements with a temperature increase of more than 10 K the time necessary to fall below the 10 K values was evaluated.

### Statistical analysis

Statistical evaluation was performed using Microsoft Excel 2016 (Version 16.16.1, Microsoft Cooperation, Redmond, USA) and SPSS software package (Version 26, SPSS, Chicago, IL, USA).

The parameters were each determined using a Man-Whitney-U test or a Kruskal-Wallis). Significance level was set at *p* = 0.05 for all statistical tests.

## Results

A summary of all results is given in Table 1.

**Table 1:**
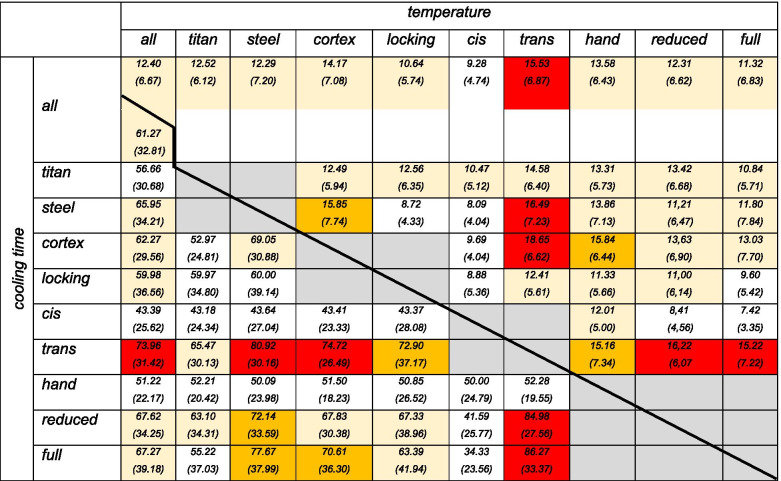
Summary of results as mean temperature increase [K] with (SD) and mean time [sec] with (SD) necessary to fall below the critical 10 K temperature increase. All values with more than 10 K temperature increase or more than 60 s. to fall below critical 10 K temperature value are marked light yellow. All values with more than 15 K increase or mor than 70 s. were marked in dark yellow. In red all critical values are marked (combination of more than 15 K heating AND more than 70 s. cooling time)

### Bone mineral density

Mean bone mineral Density was 1125.13 mgHA/ccm (Range from 932.65 to 1221.65 mgHA/ccm, SD 95.0 mgHA/ccm).

### General heating

The average heating over all measurements was 12.04 K (SD 6.67 K), the mean value for the mTCb is 0.159 K/g bone (SD 0.86). The mean time necessary to fall below the critical 10 K values was 61.27 s (SD 32.81).

### Screw-material

**Fig. 4:**
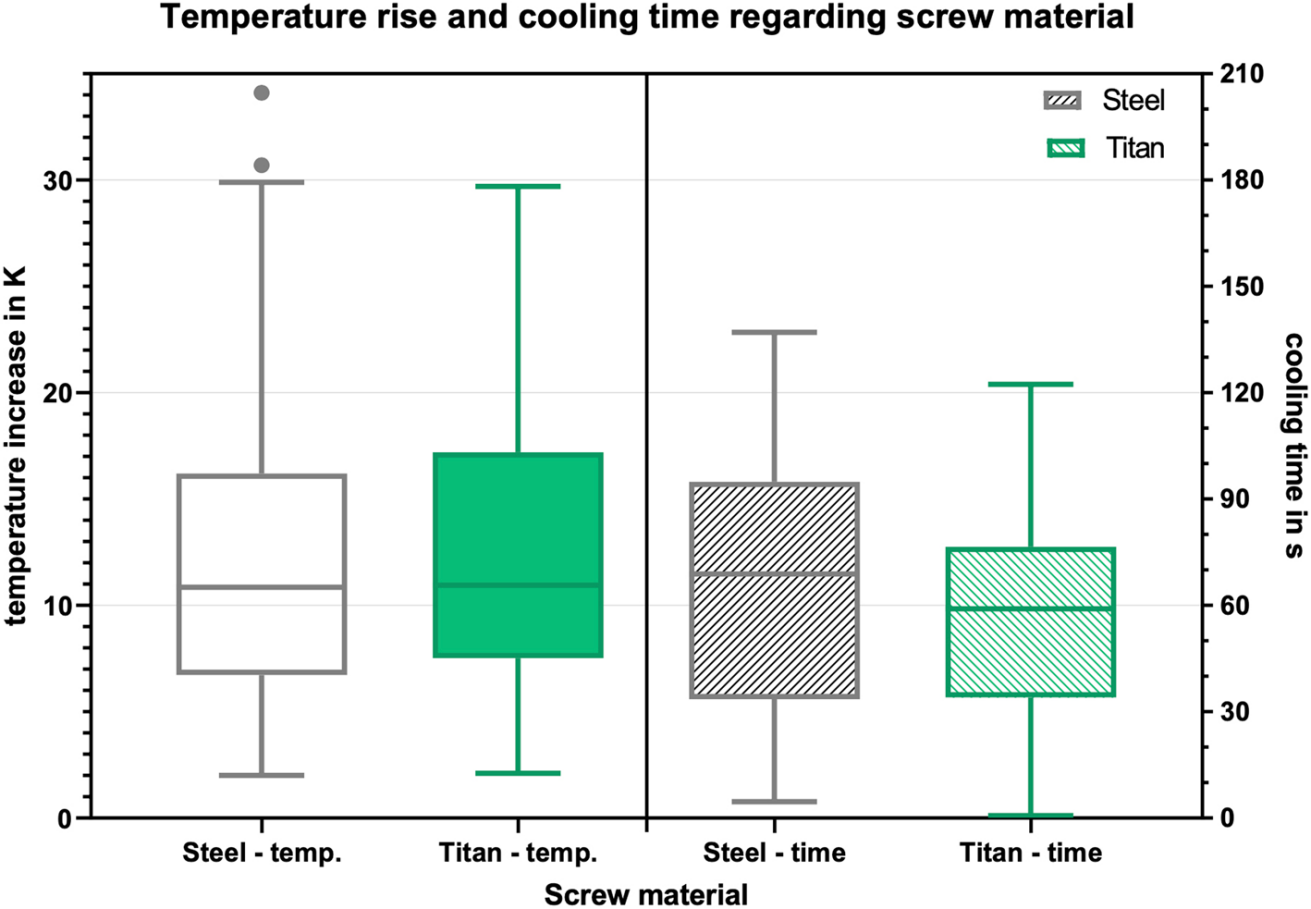
Box-plot diagram of the temperature increase and cooling time for both screw materials

No significant difference in temperature development was seen when comparing steel and titanium screws (Fig. 4, *p* = 0.447 for delta K and *p* = 0.295 for the mTCb). The cooling time was significant longer for the steel screws (*p* = 0.013). This is especially the case with the combination of steel and cortex screw (steel+cortex: 69 s. vs. steel+locking: 60 s., *p* = 0.289).

### Screw-design

**Fig. 5:**
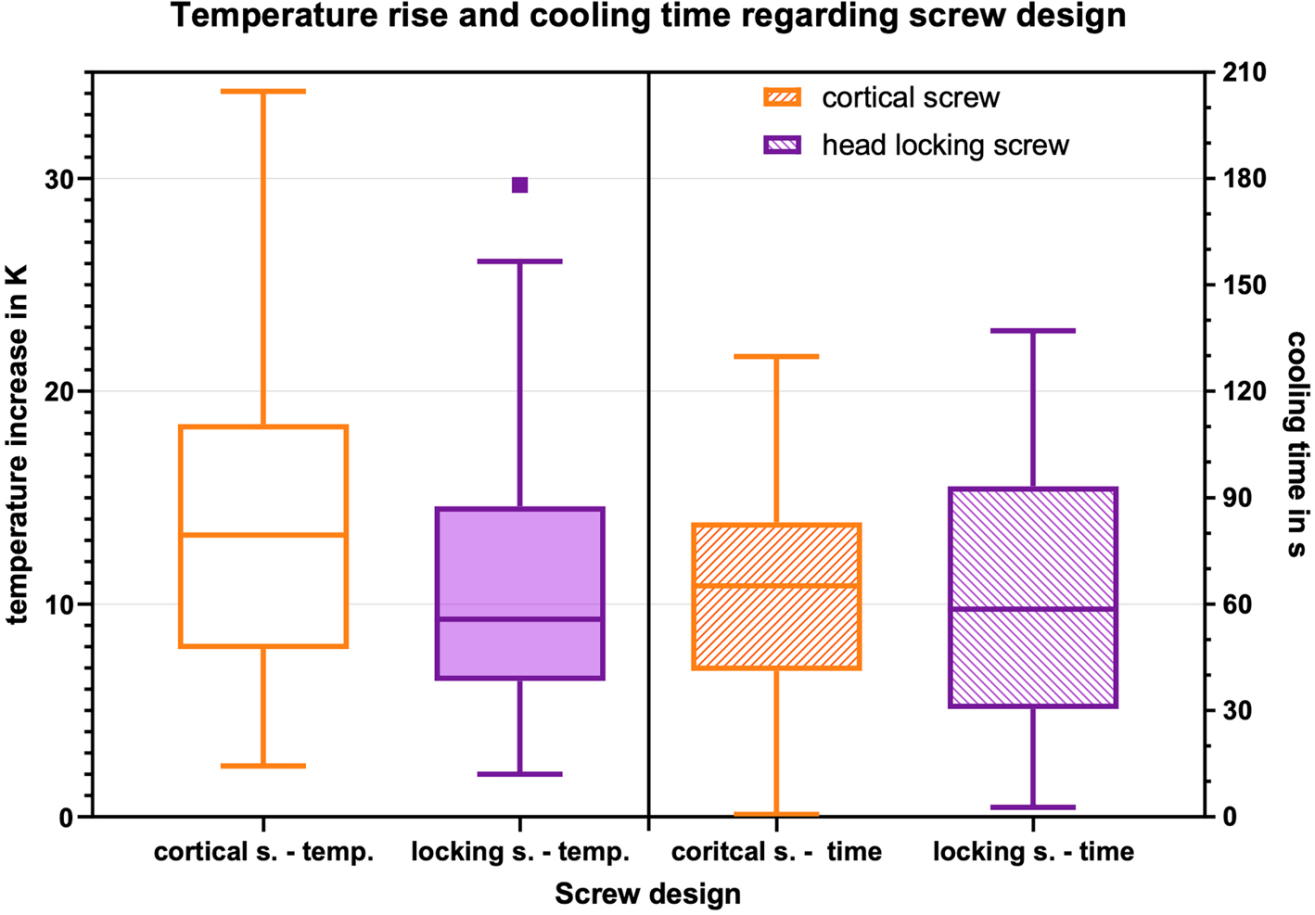
Box-plot diagram of the temperature increase and cooling time for both screw designs

When comparing cortical and head locking screws, a significant higher temperature increase for the cortical screw design could be found (*p* = 0.000 for delta K and *p* = 0.002 for the mTCb). (Fig. 5). There was no significant difference in the cooling time (*p* = 0.599).

### Location of measurement

**Fig. 6:**
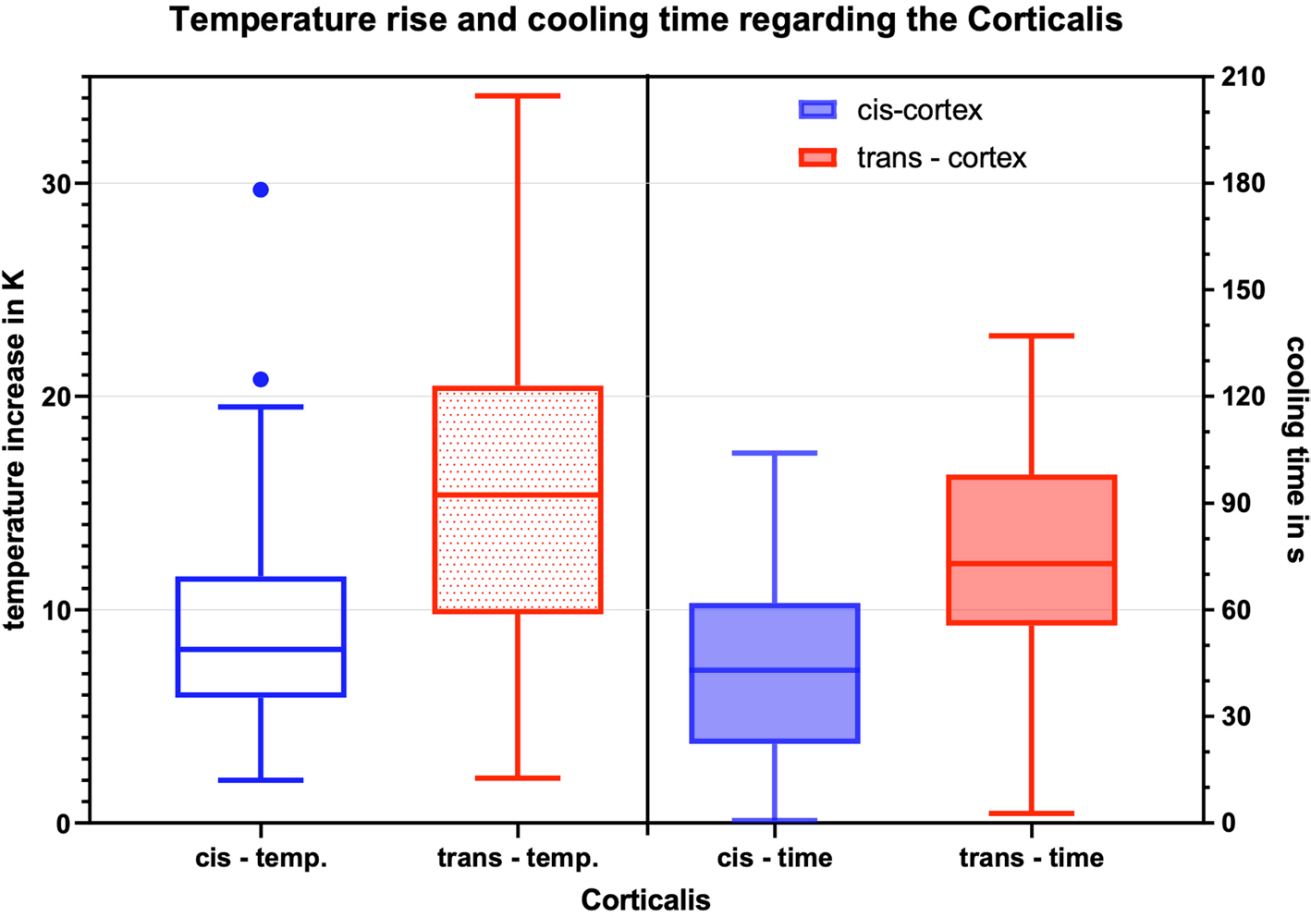
Box-plot diagram of the temperature increase and cooling time at the cis- and trans-cortex for all measurements

On average, the temperature sensors on the trans-cortex heated up more than those on the cis-cortex (*p* = 0.000 for both delta K and mTCb, Fig. 6). Additionally, the cooling time is significant longer for the trans-cortex (*p* = 0.000).

### Mechanism of screw insertion

**Fig. 7:**
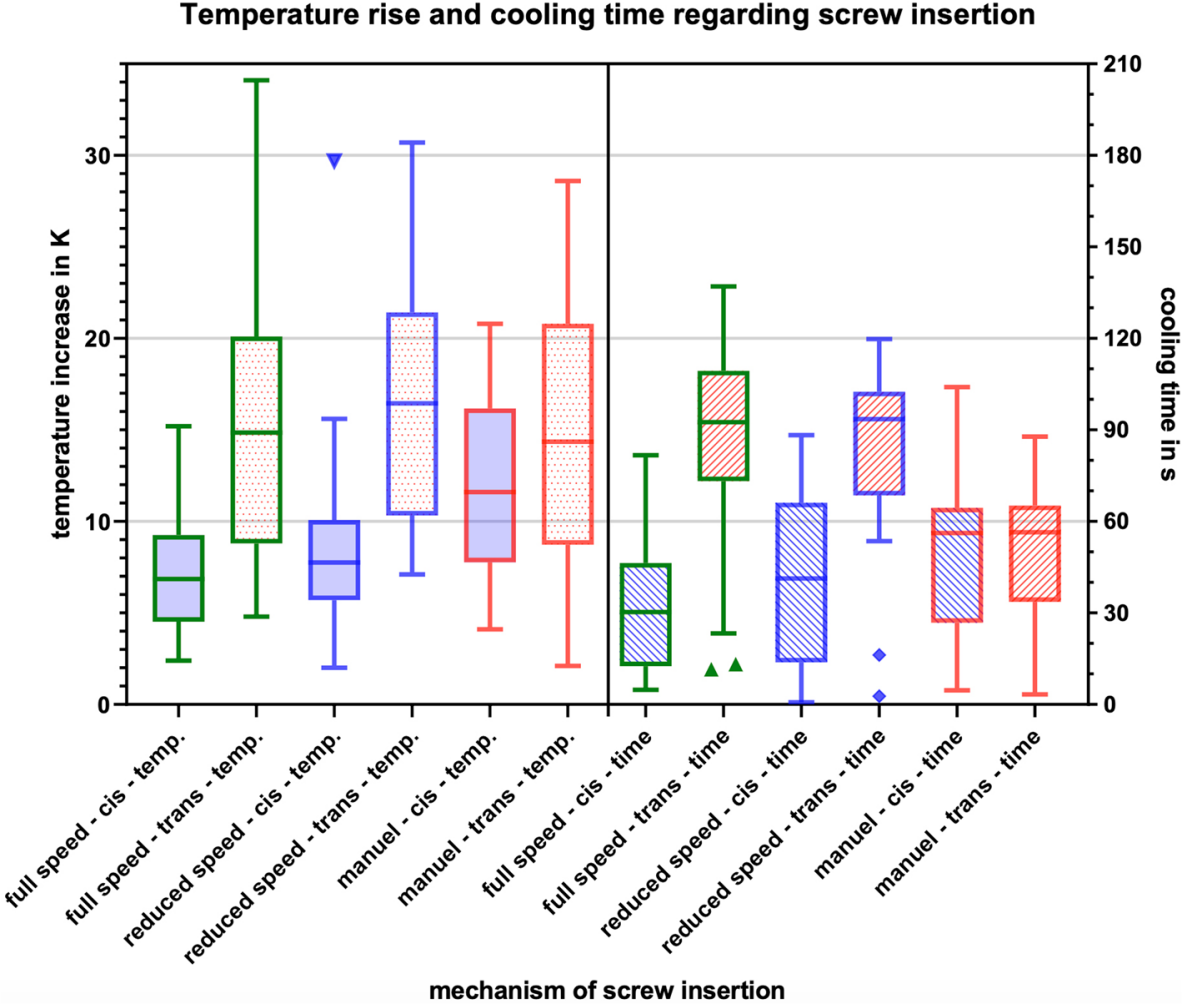
Box-plot diagram of the temperature increase and cooling time at different mechanisms of screw insertion

Manual screw insertion was associated with the highest heat generation followed by machine screw insertion at reduced speed (*p* = 0.541) and full speed (*p* = 0.021, Fig. 7). There was no statistical difference between full and reduced speed machine insertion (*p* = 0.549).

However, manual screw insertion showed similar increase at cis- and trans-cortex (*p* = 0.645), whereas both machine insertions showed significant higher increase at the trans-cortex (reduced speed: *p* = 0.000, full speed: p = 0.000). The same is true for the consideration of the coefficient instead of the temperature in K.

Regarding the cooling time manual insertion showed similar times at the cis- and trans-cortex (below 60 s.), whereas both types of machine insertion showed significant longer cooling times at the trans-cortex (reduced speed: *p* = 0.000, full speed: p = 0.000).

### Critical combination (> 15 K heating AND > 70 s. cooling)

The first thing to note is that the combination of these critical values occurs only at the trans-cortex. Parameters that lead to these critical values being exceeded are the screw material steel, the cortical screw design and the insertion of the screw with the machine both at reduced and full speed.

## Discussion

During drilling and also during screw insertion, the resistance of the cortical bone causes an increase of the temperature in the surrounding bone tissue. The main cause for the temperature increase is frictional heat [11–14]. Several parameters have already been identified for drilling that have an influence on temperature development. For example, Chen et al. were able to show that the temperature increases with the thickness of the cortical bone, the bone density and the drill diameter [15].

In order to answer the question of whether osteonecrotic temperatures occur while screw insertion, the hypothesis must first be made that the temperature difference achieved at room temperature would be constant at body temperature conditions (37 °C). Assuming normal body temperature conditions of 37 °C for the bone in vivo and considering the osteonecrotic temperature limit of 47 °C set by Eriksson et al. [3], osteonecrosis would already occur from a temperature difference of 10 °C. Under this assumption, 134 of the 240 measurements (trans- and cis-cortex considered separately) would have exceeded or at least reached osteonecrotic temperatures (> 47 °C). In addition to the absolute temperature increase, a second parameter, the duration of the critical temperature rise, is important for the development of osteonecrosis and must be considered [3–5].

In a preliminary test, we were unable to determine any significant influence of a second use of the screw (cortex and locking screw) with regard to an increased temperature development. This might be important also for intraoperative application. The surgeon can replace a screw and reuse it at another location without having to fear an increased risk of thermal induced osteonecrosis.

Our investigation showed that the screw material (titanium alloy and stainless steel) has no significant influence on the temperature development, but steel screws showed a significant longer cooling time. A possible reason for this result could be the different properties of the materials in terms of thermal conductivity. Due to their better thermal conductivity, steel screws absorb and release more heat. Titanium screws, on the other hand, absorb less heat due to their lower thermal conductivity and can therefore also release less heat.

However, the screw design has significant impact on the heat generation. Higher temperatures were achieved by the cortical screw design compared to the head locking screw design. The cortical screw design has a larger surface area, which would actually favor heat dissipation, but it has only about 60% of the mass of the head locking screw design. Thus, the heat can be dissipated less into the screw and remains in the bone.

Additionally, the trans-cortex showed significantly higher temperatures over all measurements compared to the cis-cortex. Furthermore, the cooling time was significantly longer for the trans-cortex, too. It can be assumed that the greatest frictional forces occur at the screw tip (thread cutting) and accumulate in the trans-cortex. The heat is then transmitted to the bone tissue of the trans-cortex and leads to a significantly higher heating. Additional attention must be given to the medullary canal. In the present study, this was no longer completely filled with medullary material due to sample preparation. This influences the temperature development and dissipation.

We can identify some parameters that lead to a critical combination at the trans-cortex (> 15 K temperature rise and > 70 s. cooling time). The screw material steel, the screw design cortex screw and the insertion of the screw with machine at reduced and full speed are to be regarded as unfavorable.

An investigation by Manoogian et al. confirmed the significant influence of the screw design. They investigated the heat generation the during insertion of standard and self-drilling Schanz-Pins into mid-diaphysis bovine femora. This group showed the highest temperature rise for the self-drilling pins followed by the insertion of self-drilling pins into a pre-drilled hole. The lowest rise was found for the insertion of standard pins into a predrilled hole [16].

The mechanism of screw insertion also seems to have a substantial impact on the heat generation. The highest temperature rise was found when the screws were inserted by hand, as opposed to the lowest in the group using full speed machine insertion. But, the cooling time shows uncritical values for the manual screw insertion. The relatively high temperature increase can be explained by an increase in both the cis- and the trans-cortex (whereas when screwing with the machine, only the opposite cortex shows a relevant temperature increase). Thus, manual screw insertion can be defined as safe method of screw insertion.

Both steps of placing a screw, the drilling and the screw insertion, have the potential to increase temperature of the surrounding bone tissue above critical values and therefore can induce osteonecrosis. This can finally result in screw loosening or ultimate failure of the osteosynthesis [3, 16, 17]. The trans-cortex in particular is affected by a critical increase in temperature and a long cooling time.

One effective strategy to prevent osteonecrosis during drilling is sufficient irrigation. Sener at al. investigated the effects of irrigation on heat generation in different drilling depths. Interestingly, they found the highest rise in temperature without irrigation in a depth of 3 mm. With irrigation the highest temperature was found in 12 mm depth, but the temperature maximum was only 36.4 °C. Using saline solution with a temperature of 10 °C all measurements were below body temperature [18]. This technique only allows cooling around the entry point when screwing in screws. Therefore, the effectiveness especially in the trans-cortex region is limited. It would be conceivable to irrigate the screw holes with cold saline before inserting the screw or to cool down the screw in a cold saline bath prior to insertion. Recently, Bruketa et al. described a method that allows cooling during drilling by using a cannulated drill bit perforated at the tip in combination with flow rate control. The group was able to demonstrate that the application is possible without increasing intramedullary pressure. This system provides a means of cooling the drill bit and the bone. However, the efficiency of the cooling was not investigated in this work [19]. In addition, it is known from studies by Haddad et al. that cooling bones prior to deburring has a tendency to increase the fusion rate and bone strength after the healing process. It is therefore questionable whether cooling not only limits damage but also promotes bone healing [20].

This study also has limitations. The experiments have been performed at room temperature and not at body temperature. This was done in accordance with the study of Matthews and Hirsch, who found no difference in the temperature rise during bone drilling in in vivo and in in vitro [8]. Additionally, we used standardized laboratory settings for drilling and screw insertion to minimize confounding co-factors. This does not fully represent the intraoperative setting in an operation theater.

## Conclusion

Both steps of placing a screw, drilling and screw insertion, have the potential to increase the temperature of the surrounding bone tissue above critical values and therefore can induce osteonecrosis.

Relevant influence on temperature development is the screw design (cortical > locking), the location of measurement (trans-cortex > cis-cortex) and the mode of screw insertion (hand insertion > machine insertion).

Relevant influence on cooling time have the screw material (steel > titanium), the location of measurement (trans-cortex > cis-cortex) and the mode of screw insertion (machine insertion > hand insertion).

Critical combination of both parameters (> 15 K heating AND > 70 s. cooling) can only be found at the trans-cortex. Parameters that lead to these critical values being exceeded are the screw material steel, the cortical screw design and the screw insertion with the machine both at reduced and full speed.

The screw design and the location of measurement cannot directly be influenced. The higher temperature development at the trans-cortex makes effective irrigation problematic. It would be conceivable to cool the borehole with cold saline solution before inserting the screw or to cool the screw in cold saline solution.

The surgeon, however, should be aware that the technique of insertion of the fixation elements may influence the later outcome of the entire construct. If possible, insertion by hand and irrigation should be considered.

## Data Availability

The datasets used and/or analysed during the current study are available from the corresponding author on reasonable request.

## Abbreviations

°C: degree Celcius
(v)BMD: (volumetric) Bone Mineral Density
ccm: Cubic centimeter
CT: Computer tomography
HU: Hounsfield unit
K: Kelvin
mgHA: milligram Hydroxyapatite
mTCb: Modified temperature coefficient for bones
pQCT: Peripher quantitative computer tomography
ROI: Region of interest
rpm: Rounds per minute
